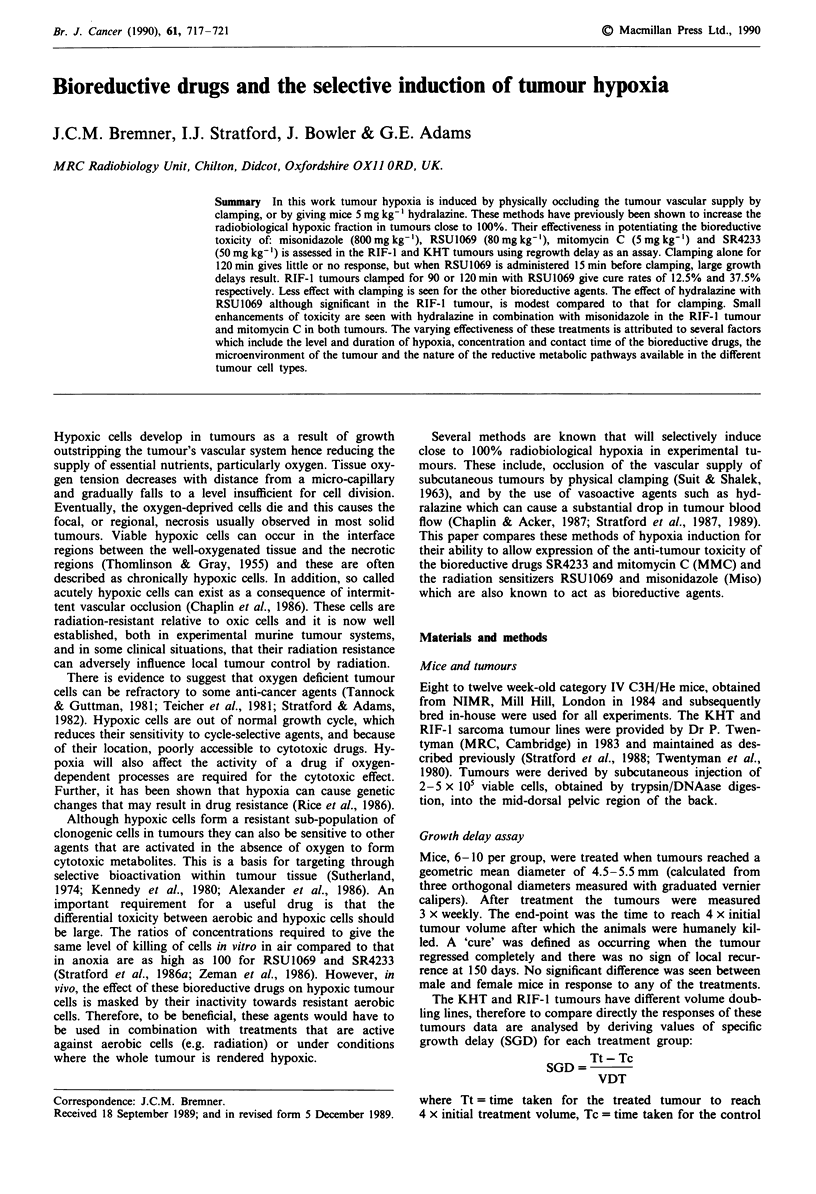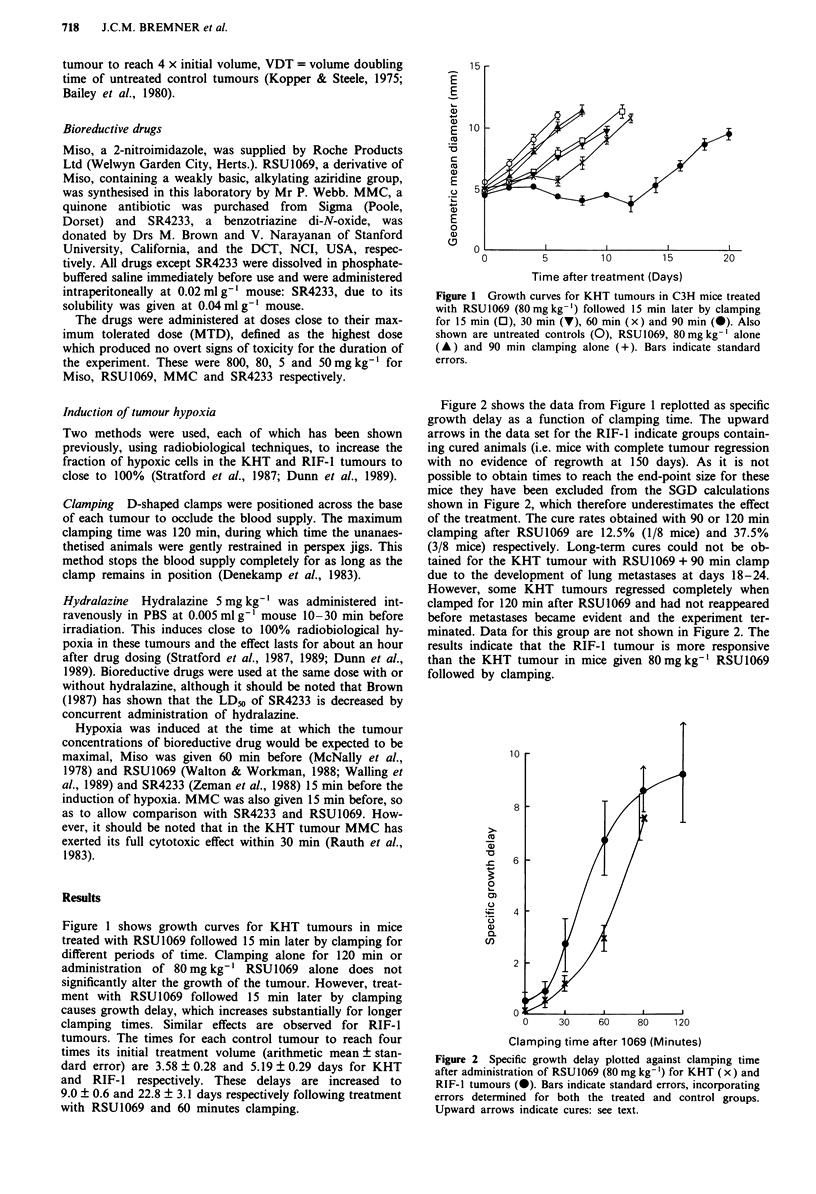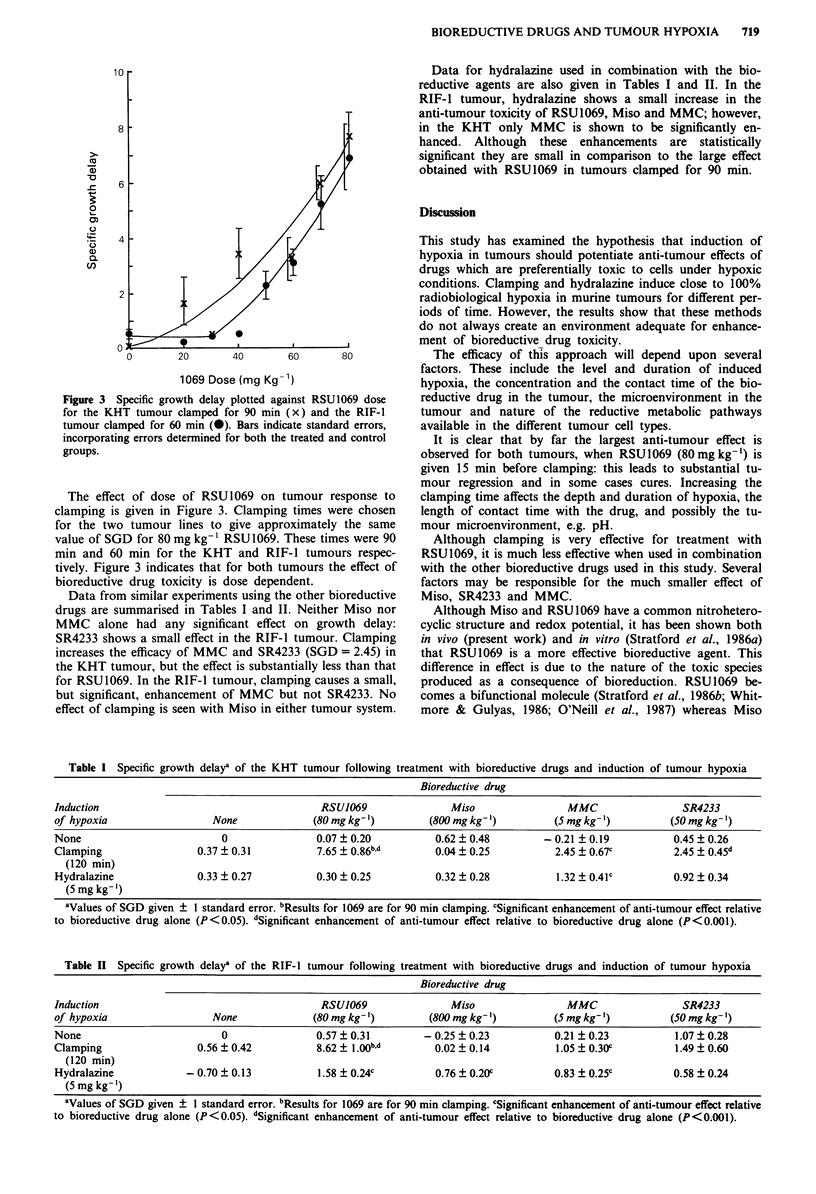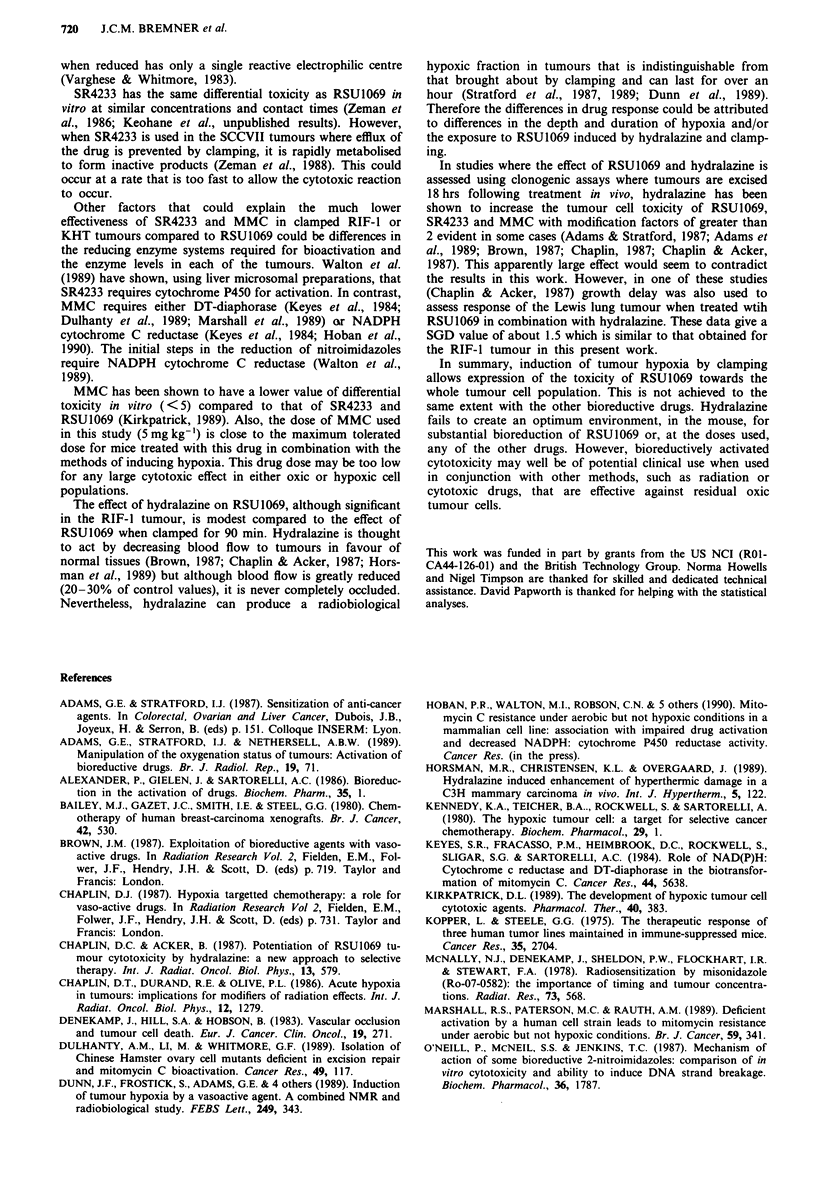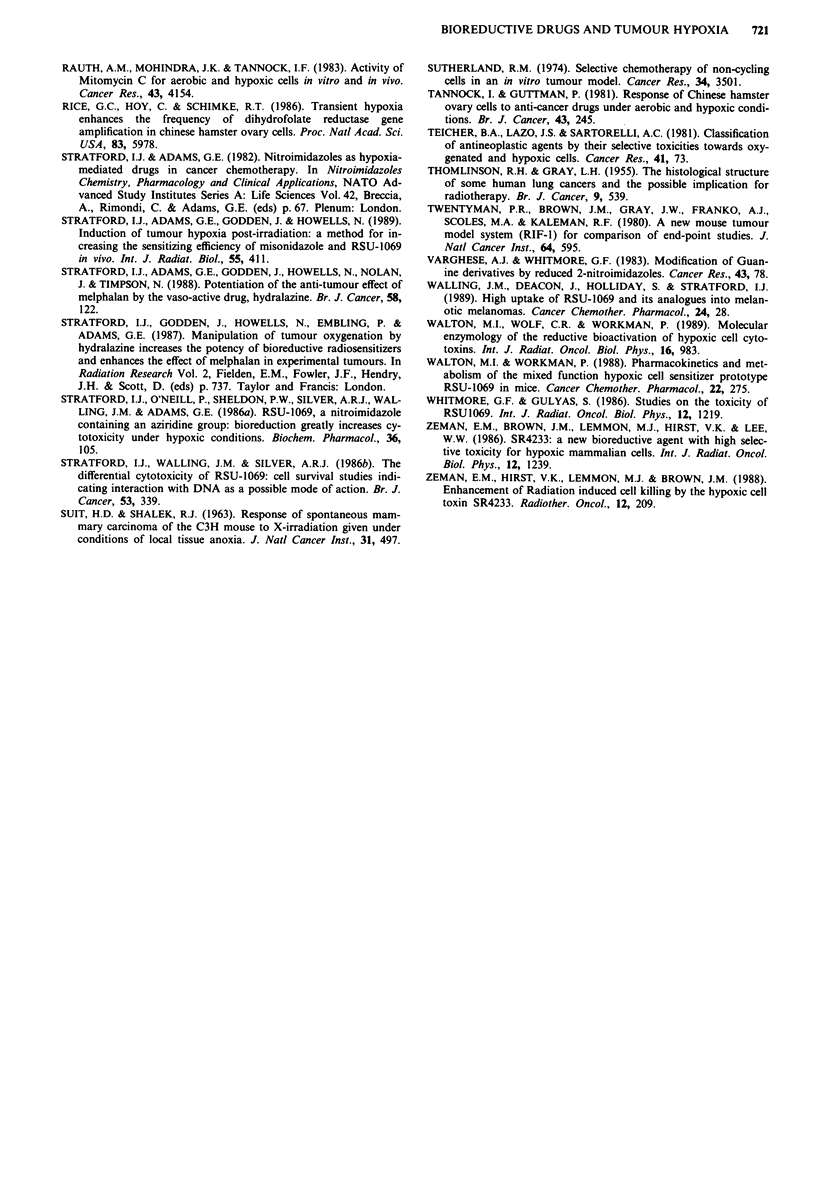# Bioreductive drugs and the selective induction of tumour hypoxia.

**DOI:** 10.1038/bjc.1990.161

**Published:** 1990-05

**Authors:** J. C. Bremner, I. J. Stratford, J. Bowler, G. E. Adams

**Affiliations:** MRC Radiobiology Unit, Chilton, Didcot, Oxfordshire, UK.

## Abstract

In this work tumour hypoxia is induced by physically occluding the tumour vascular supply by clamping, or by giving mice 5 mg kg-1 hydralazine. These methods have previously been shown to increase the radiobiological hypoxic fraction in tumours close to 100%. Their effectiveness in potentiating the bioreductive toxicity of: misonidazole (800 mg kg-1), RSU1069 (80 mg kg-1), mitomycin C (5 mg kg-1) and SR4233 (50 mg kg-1) is assessed in the RIF-1 and KHT tumours using regrowth delay as an assay. Clamping alone for 120 min gives little or no response, but when RSU1069 is administered 15 min before clamping, large growth delays result. RIF-1 tumours clamped for 90 or 120 min with RSU1069 give cure rates of 12.5% and 37.5% respectively. Less effect with clamping is seen for the other bioreductive agents. The effect of hydralazine with RSU1069 although significant in the RIF-1 tumour, is modest compared to that for clamping. Small enhancements of toxicity are seen with hydralazine in combination with misonidazole in the RIF-1 tumour and mitomycin C in both tumours. The varying effectiveness of these treatments is attributed to several factors which include the level and duration of hypoxia, concentration and contact time of the bioreductive drugs, the microenvironment of the tumour and the nature of the reductive metabolic pathways available in the different tumour cell types.


Br. J. Cancer (1990), 61, 717-721 © Macmillan Press Ltd., 1990

### Bioreductive drugs and the selective induction of tumour hypoxia

J.C.M. Bremner, I.J. Stratford, J. Bowler & G.E. Adams

*MRC Radiobiology Unit, Chilton, Didcot, Oxfordshire OX11 0RD, UK.*

**Summary** In this work tumour hypoxia is induced by physically occluding the tumour vascular supply by clamping, or by giving mice 5 mg $kg^{-1}$ hydralazine. These methods have previously been shown to increase the radiobiological hypoxic fraction in tumours close to 100%. Their effectiveness in potentiating the bioreductive toxicity of: misonidazole (800 mg $kg^{-1}$), RSU1069 (80 mg $kg^{-1}$), mitomycin C (5 mg $kg^{-1}$) and SR4233 (50 mg $kg^{-1}$) is assessed in the RIF-1 and KHT tumours using regrowth delay as an assay. Clamping alone for 120 min gives little or no response, but when RSU1069 is administered 15 min before clamping, large growth delays result. RIF-1 tumours clamped for 90 or 120 min with RSU1069 give cure rates of 12.5% and 37.5% respectively. Less effect with clamping is seen for the other bioreductive agents. The effect of hydralazine with RSU1069 although significant in the RIF-1 tumour, is modest compared to that for clamping. Small enhancements of toxicity are seen with hydralazine in combination with misonidazole in the RIF-1 tumour and mitomycin C in both tumours. The varying effectiveness of these treatments is attributed to several factors which include the level and duration of hypoxia, concentration and contact time of the bioreductive drugs, the microenvironment of the tumour and the nature of the reductive metabolic pathways available in the different tumour cell types.

Hypoxic cells develop in tumours as a result of growth outstripping the tumour's vascular system hence reducing the supply of essential nutrients, particularly oxygen. Tissue oxygen tension decreases with distance from a micro-capillary and gradually falls to a level insufficient for cell division. Eventually, the oxygen-deprived cells die and this causes the focal, or regional, necrosis usually observed in most solid tumours. Viable hypoxic cells can occur in the interface regions between the well-oxygenated tissue and the necrotic regions (Thomlinson & Gray, 1955) and these are often described as chronically hypoxic cells. In addition, so called acutely hypoxic cells can exist as a consequence of intermittent vascular occlusion (Chaplin *et al.*, 1986). These cells are radiation-resistant relative to oxic cells and it is now well established, both in experimental murine tumour systems, and in some clinical situations, that their radiation resistance can adversely influence local tumour control by radiation.

There is evidence to suggest that oxygen deficient tumour cells can be refractory to some anti-cancer agents (Tannock & Guttman, 1981; Teicher *et al.*, 1981; Stratford & Adams, 1982). Hypoxic cells are out of normal growth cycle, which reduces their sensitivity to cycle-selective agents, and because of their location, poorly accessible to cytotoxic drugs. Hypoxia will also affect the activity of a drug if oxygen-dependent processes are required for the cytotoxic effect. Further, it has been shown that hypoxia can cause genetic changes that may result in drug resistance (Rice *et al.*, 1986).

Although hypoxic cells form a resistant sub-population of clonogenic cells in tumours they can also be sensitive to other agents that are activated in the absence of oxygen to form cytotoxic metabolites. This is a basis for targeting through selective bioactivation within tumour tissue (Sutherland, 1974; Kennedy *et al.*, 1980; Alexander *et al.*, 1986). An important requirement for a useful drug is that the differential toxicity between aerobic and hypoxic cells should be large. The ratios of concentrations required to give the same level of killing of cells *in vitro* in air compared to that in anoxia are as high as 100 for RSU1069 and SR4233 (Stratford *et al.*, 1986*a*; Zeman *et al.*, 1986). However, *in vivo*, the effect of these bioreductive drugs on hypoxic tumour cells is masked by their inactivity towards resistant aerobic cells. Therefore, to be beneficial, these agents would have to be used in combination with treatments that are active against aerobic cells (e.g. radiation) or under conditions where the whole tumour is rendered hypoxic.

Correspondence: J.C.M. Bremner.
Received 18 September 1989; and in revised form 5 December 1989.

Several methods are known that will selectively induce close to 100% radiobiological hypoxia in experimental tumours. These include, occlusion of the vascular supply of subcutaneous tumours by physical clamping (Suit & Shalek, 1963), and by the use of vasoactive agents such as hydralazine which can cause a substantial drop in tumour blood flow (Chaplin & Acker, 1987; Stratford *et al.*, 1987, 1989). This paper compares these methods of hypoxia induction for their ability to allow expression of the anti-tumour toxicity of the bioreductive drugs SR4233 and mitomycin C (MMC) and the radiation sensitizers RSU1069 and misonidazole (Miso) which are also known to act as bioreductive agents.

### Materials and methods

### Mice and tumours

Eight to twelve week-old category IV C3H/He mice, obtained from NIMR, Mill Hill, London in 1984 and subsequently bred in-house were used for all experiments. The KHT and RIF-1 sarcoma tumour lines were provided by Dr P. Twentyman (MRC, Cambridge) in 1983 and maintained as described previously (Stratford *et al.*, 1988; Twentyman *et al.*, 1980). Tumours were derived by subcutaneous injection of $2-5 \times 10^5$ viable cells, obtained by trypsin/DNAase digestion, into the mid-dorsal pelvic region of the back.

### Growth delay assay

Mice, 6-10 per group, were treated when tumours reached a geometric mean diameter of 4.5-5.5 mm (calculated from three orthogonal diameters measured with graduated vernier calipers). After treatment the tumours were measured 3 × weekly. The end-point was the time to reach 4 × initial tumour volume after which the animals were humanely killed. A 'cure' was defined as occurring when the tumour regressed completely and there was no sign of local recurrence at 150 days. No significant difference was seen between male and female mice in response to any of the treatments.

The KHT and RIF-1 tumours have different volume doubling lines, therefore to compare directly the responses of these tumours data are analysed by deriving values of specific growth delay (SGD) for each treatment group:

$$SGD = \frac{Tt - Tc}{VDT}$$

where Tt = time taken for the treated tumour to reach 4 × initial treatment volume, Tc = time taken for the control tumour to reach 4 × initial volume, VDT = volume doubling time of untreated control tumours (Kopper & Steele, 1975; Bailey *et al.*, 1980).

*Bioreductive drugs*

Miso, a 2-nitroimidazole, was supplied by Roche Products Ltd (Welwyn Garden City, Herts.). RSU1069, a derivative of Miso, containing a weakly basic, alkylating aziridine group, was synthesised in this laboratory by Mr P. Webb. MMC, a quinone antibiotic was purchased from Sigma (Poole, Dorset) and SR4233, a benzotriazine di-*N*-oxide, was donated by Drs M. Brown and V. Narayanan of Stanford University, California, and the DCT, NCI, USA, respectively. All drugs except SR4233 were dissolved in phosphate-buffered saline immediately before use and were administered intraperitoneally at 0.02 ml $g^{-1}$ mouse: SR4233, due to its solubility was given at 0.04 ml $g^{-1}$ mouse.

The drugs were administered at doses close to their maximum tolerated dose (MTD), defined as the highest dose which produced no overt signs of toxicity for the duration of the experiment. These were 800, 80, 5 and 50 mg $kg^{-1}$ for Miso, RSU1069, MMC and SR4233 respectively.

*Induction of tumour hypoxia*

Two methods were used, each of which has been shown previously, using radiobiological techniques, to increase the fraction of hypoxic cells in the KHT and RIF-1 tumours to close to 100% (Stratford *et al.*, 1987; Dunn *et al.*, 1989).

*Clamping* D-shaped clamps were positioned across the base of each tumour to occlude the blood supply. The maximum clamping time was 120 min, during which time the unanaesthetised animals were gently restrained in perspex jigs. This method stops the blood supply completely for as long as the clamp remains in position (Denekamp *et al.*, 1983).

*Hydralazine* Hydralazine 5 mg $kg^{-1}$ was administered intravenously in PBS at 0.005 ml $g^{-1}$ mouse 10–30 min before irradiation. This induces close to 100% radiobiological hypoxia in these tumours and the effect lasts for about an hour after drug dosing (Stratford *et al.*, 1987, 1989; Dunn *et al.*, 1989). Bioreductive drugs were used at the same dose with or without hydralazine, although it should be noted that Brown (1987) has shown that the $LD_{50}$ of SR4233 is decreased by concurrent administration of hydralazine.

Hypoxia was induced at the time at which the tumour concentrations of bioreductive drug would be expected to be maximal, Miso was given 60 min before (McNally *et al.*, 1978) and RSU1069 (Walton & Workman, 1988; Walling *et al.*, 1989) and SR4233 (Zeman *et al.*, 1988) 15 min before the induction of hypoxia. MMC was also given 15 min before, so as to allow comparison with SR4233 and RSU1069. However, it should be noted that in the KHT tumour MMC has exerted its full cytotoxic effect within 30 min (Rauth *et al.*, 1983).

### Results

Figure 1 shows growth curves for KHT tumours in mice treated with RSU1069 followed 15 min later by clamping for different periods of time. Clamping alone for 120 min or administration of 80 mg $kg^{-1}$ RSU1069 alone does not significantly alter the growth of the tumour. However, treatment with RSU1069 followed 15 min later by clamping causes growth delay, which increases substantially for longer clamping times. Similar effects are observed for RIF-1 tumours. The times for each control tumour to reach four times its initial treatment volume (arithmetic mean ± standard error) are 3.58 ± 0.28 and 5.19 ± 0.29 days for KHT and RIF-1 respectively. These delays are increased to 9.0 ± 0.6 and 22.8 ± 3.1 days respectively following treatment with RSU1069 and 60 minutes clamping.

**Figure 1** Growth curves for KHT tumours in C3H mice treated with RSU1069 (80 mg $kg^{-1}$) followed 15 min later by clamping for 15 min (□), 30 min (▼), 60 min (×) and 90 min (●). Also shown are untreated controls (O), RSU1069, 80 mg $kg^{-1}$ alone (▲) and 90 min clamping alone (+). Bars indicate standard errors.

Figure 2 shows the data from Figure 1 replotted as specific growth delay as a function of clamping time. The upward arrows in the data set for the RIF-1 indicate groups containing cured animals (i.e. mice with complete tumour regression with no evidence of regrowth at 150 days). As it is not possible to obtain times to reach the end-point size for these mice they have been excluded from the SGD calculations shown in Figure 2, which therefore underestimates the effect of the treatment. The cure rates obtained with 90 or 120 min clamping after RSU1069 are 12.5% (1/8 mice) and 37.5% (3/8 mice) respectively. Long-term cures could not be obtained for the KHT tumour with RSU1069 + 90 min clamp due to the development of lung metastases at days 18–24. However, some KHT tumours regressed completely when clamped for 120 min after RSU1069 and had not reappeared before metastases became evident and the experiment terminated. Data for this group are not shown in Figure 2. The results indicate that the RIF-1 tumour is more responsive than the KHT tumour in mice given 80 mg $kg^{-1}$ RSU1069 followed by clamping.

**Figure 2** Specific growth delay plotted against clamping time after administration of RSU1069 (80 mg $kg^{-1}$) for KHT (×) and RIF-1 tumours (●). Bars indicate standard errors, incorporating errors determined for both the treated and control groups. Upward arrows indicate cures: see text.

**Figure 3** Specific growth delay plotted against RSU1069 dose for the KHT tumour clamped for 90 min (×) and the RIF-1 tumour clamped for 60 min (●). Bars indicate standard errors, incorporating errors determined for both the treated and control groups.

The effect of dose of RSU1069 on tumour response to clamping is given in Figure 3. Clamping times were chosen for the two tumour lines to give approximately the same value of SGD for 80 mg kg$^{-1}$ RSU1069. These times were 90 min and 60 min for the KHT and RIF-1 tumours respectively. Figure 3 indicates that for both tumours the effect of bioreductive drug toxicity is dose dependent.

Data from similar experiments using the other bioreductive drugs are summarised in Tables I and II. Neither Miso nor MMC alone had any significant effect on growth delay: SR4233 shows a small effect in the RIF-1 tumour. Clamping increases the efficacy of MMC and SR4233 (SGD = 2.45) in the KHT tumour, but the effect is substantially less than that for RSU1069. In the RIF-1 tumour, clamping causes a small, but significant, enhancement of MMC but not SR4233. No effect of clamping is seen with Miso in either tumour system.

Data for hydralazine used in combination with the bioreductive agents are also given in Tables I and II. In the RIF-1 tumour, hydralazine shows a small increase in the anti-tumour toxicity of RSU1069, Miso and MMC; however, in the KHT only MMC is shown to be significantly enhanced. Although these enhancements are statistically significant they are small in comparison to the large effect obtained with RSU1069 in tumours clamped for 90 min.

### Discussion

This study has examined the hypothesis that induction of hypoxia in tumours should potentiate anti-tumour effects of drugs which are preferentially toxic to cells under hypoxic conditions. Clamping and hydralazine induce close to 100% radiobiological hypoxia in murine tumours for different periods of time. However, the results show that these methods do not always create an environment adequate for enhancement of bioreductive drug toxicity.

The efficacy of this approach will depend upon several factors. These include the level and duration of induced hypoxia, the concentration and the contact time of the bioreductive drug in the tumour, the microenvironment in the tumour and nature of the reductive metabolic pathways available in the different tumour cell types.

It is clear that by far the largest anti-tumour effect is observed for both tumours, when RSU1069 (80 mg kg$^{-1}$) is given 15 min before clamping: this leads to substantial tumour regression and in some cases cures. Increasing the clamping time affects the depth and duration of hypoxia, the length of contact time with the drug, and possibly the tumour microenvironment, e.g. pH.

Although clamping is very effective for treatment with RSU1069, it is much less effective when used in combination with the other bioreductive drugs used in this study. Several factors may be responsible for the much smaller effect of Miso, SR4233 and MMC.

Although Miso and RSU1069 have a common nitroheterocyclic structure and redox potential, it has been shown both *in vivo* (present work) and *in vitro* (Stratford *et al.*, 1986*a*) that RSU1069 is a more effective bioreductive agent. This difference in effect is due to the nature of the toxic species produced as a consequence of bioreduction. RSU1069 becomes a bifunctional molecule (Stratford *et al.*, 1986*b*; Whitmore & Gulyas, 1986; O'Neill *et al.*, 1987) whereas Miso

**Table I** Specific growth delay[a] of the KHT tumour following treatment with bioreductive drugs and induction of tumour hypoxia

| Induction of hypoxia | None | Bioreductive drug: RSU1069 (80 mg kg$^{-1}$) | Miso (800 mg kg$^{-1}$) | MMC (5 mg kg$^{-1}$) | SR4233 (50 mg kg$^{-1}$) |
|---|---|---|---|---|---|
| None | 0 | 0.07 ± 0.20 | 0.62 ± 0.48 | − 0.21 ± 0.19 | 0.45 ± 0.26 |
| Clamping (120 min) | 0.37 ± 0.31 | 7.65 ± 0.86[b,d] | 0.04 ± 0.25 | 2.45 ± 0.67[c] | 2.45 ± 0.45[d] |
| Hydralazine (5 mg kg$^{-1}$) | 0.33 ± 0.27 | 0.30 ± 0.25 | 0.32 ± 0.28 | 1.32 ± 0.41[c] | 0.92 ± 0.34 |

[a]Values of SGD given ± 1 standard error. [b]Results for 1069 are for 90 min clamping. [c]Significant enhancement of anti-tumour effect relative to bioreductive drug alone ($P<0.05$). [d]Significant enhancement of anti-tumour effect relative to bioreductive drug alone ($P<0.001$).

**Table II** Specific growth delay[a] of the RIF-1 tumour following treatment with bioreductive drugs and induction of tumour hypoxia

| Induction of hypoxia | None | Bioreductive drug: RSU1069 (80 mg kg$^{-1}$) | Miso (800 mg kg$^{-1}$) | MMC (5 mg kg$^{-1}$) | SR4233 (50 mg kg$^{-1}$) |
|---|---|---|---|---|---|
| None | 0 | 0.57 ± 0.31 | − 0.25 ± 0.23 | 0.21 ± 0.23 | 1.07 ± 0.28 |
| Clamping (120 min) | 0.56 ± 0.42 | 8.62 ± 1.00[b,d] | 0.02 ± 0.14 | 1.05 ± 0.30[c] | 1.49 ± 0.60 |
| Hydralazine (5 mg kg$^{-1}$) | − 0.70 ± 0.13 | 1.58 ± 0.24[c] | 0.76 ± 0.20[c] | 0.83 ± 0.25[c] | 0.58 ± 0.24 |

[a]Values of SGD given ± 1 standard error. [b]Results for 1069 are for 90 min clamping. [c]Significant enhancement of anti-tumour effect relative to bioreductive drug alone ($P<0.05$). [d]Significant enhancement of anti-tumour effect relative to bioreductive drug alone ($P<0.001$).

when reduced has only a single reactive electrophilic centre (Varghese & Whitmore, 1983).

SR4233 has the same differential toxicity as RSU1069 *in vitro* at similar concentrations and contact times (Zeman *et al.*, 1986; Keohane *et al.*, unpublished results). However, when SR4233 is used in the SCCVII tumours where efflux of the drug is prevented by clamping, it is rapidly metabolised to form inactive products (Zeman *et al.*, 1988). This could occur at a rate that is too fast to allow the cytotoxic reaction to occur.

Other factors that could explain the much lower effectiveness of SR4233 and MMC in clamped RIF-1 or KHT tumours compared to RSU1069 could be differences in the reducing enzyme systems required for bioactivation and the enzyme levels in each of the tumours. Walton *et al.* (1989) have shown, using liver microsomal preparations, that SR4233 requires cytochrome P450 for activation. In contrast, MMC requires either DT-diaphorase (Keyes *et al.*, 1984; Dulhanty *et al.*, 1989; Marshall *et al.*, 1989) or NADPH cytochrome C reductase (Keyes *et al.*, 1984; Hoban *et al.*, 1990). The initial steps in the reduction of nitroimidazoles require NADPH cytochrome C reductase (Walton *et al.*, 1989).

MMC has been shown to have a lower value of differential toxicity *in vitro* ($<5$) compared to that of SR4233 and RSU1069 (Kirkpatrick, 1989). Also, the dose of MMC used in this study ($5\ mg\ kg^{-1}$) is close to the maximum tolerated dose for mice treated with this drug in combination with the methods of inducing hypoxia. This drug dose may be too low for any large cytotoxic effect in either oxic or hypoxic cell populations.

The effect of hydralazine on RSU1069, although significant in the RIF-1 tumour, is modest compared to the effect of RSU1069 when clamped for 90 min. Hydralazine is thought to act by decreasing blood flow to tumours in favour of normal tissues (Brown, 1987; Chaplin & Acker, 1987; Horsman *et al.*, 1989) but although blood flow is greatly reduced (20–30% of control values), it is never completely occluded. Nevertheless, hydralazine can produce a radiobiological hypoxic fraction in tumours that is indistinguishable from that brought about by clamping and can last for over an hour (Stratford *et al.*, 1987, 1989; Dunn *et al.*, 1989). Therefore the differences in drug response could be attributed to differences in the depth and duration of hypoxia and/or the exposure to RSU1069 induced by hydralazine and clamping.

In studies where the effect of RSU1069 and hydralazine is assessed using clonogenic assays where tumours are excised 18 hrs following treatment *in vivo*, hydralazine has been shown to increase the tumour cell toxicity of RSU1069, SR4233 and MMC with modification factors of greater than 2 evident in some cases (Adams & Stratford, 1987; Adams *et al.*, 1989; Brown, 1987; Chaplin, 1987; Chaplin & Acker, 1987). This apparently large effect would seem to contradict the results in this work. However, in one of these studies (Chaplin & Acker, 1987) growth delay was also used to assess response of the Lewis lung tumour when treated wtih RSU1069 in combination with hydralazine. These data give a SGD value of about 1.5 which is similar to that obtained for the RIF-1 tumour in this present work.

In summary, induction of tumour hypoxia by clamping allows expression of the toxicity of RSU1069 towards the whole tumour cell population. This is not achieved to the same extent with the other bioreductive drugs. Hydralazine fails to create an optimum environment, in the mouse, for substantial bioreduction of RSU1069 or, at the doses used, any of the other drugs. However, bioreductively activated cytotoxicity may well be of potential clinical use when used in conjunction with other methods, such as radiation or cytotoxic drugs, that are effective against residual oxic tumour cells.

This work was funded in part by grants from the US NCI (R01-CA44-126-01) and the British Technology Group. Norma Howells and Nigel Timpson are thanked for skilled and dedicated technical assistance. David Papworth is thanked for helping with the statistical analyses.

### References

ADAMS, G.E. & STRATFORD, I.J. (1987). Sensitization of anti-cancer agents. In *Colorectal, Ovarian and Liver Cancer*, Dubois, J.B., Joyeux, H. & Serron, B. (eds) p. 151. Colloque INSERM: Lyon.

ADAMS, G.E., STRATFORD, I.J. & NETHERSELL, A.B.W. (1989). Manipulation of the oxygenation status of tumours: Activation of bioreductive drugs. *Br. J. Radiol. Rep.*, **19**, 71.

ALEXANDER, P., GIELEN, J. & SARTORELLI, A.C. (1986). Bioreduction in the activation of drugs. *Biochem. Pharm.*, **35**, 1.

BAILEY, M.J., GAZET, J.C., SMITH, I.E. & STEEL, G.G. (1980). Chemotherapy of human breast-carcinoma xenografts. *Br. J. Cancer*, **42**, 530.

BROWN, J.M. (1987). Exploitation of bioreductive agents with vaso-active drugs. In *Radiation Research Vol. 2*, Fielden, E.M., Folwer, J.F., Hendry, J.H. & Scott, D. (eds) p. 719. Taylor and Francis: London.

CHAPLIN, D.J. (1987). Hypoxia targetted chemotherapy: a role for vaso-active drugs. In *Radiation Research Vol 2*, Fielden, E.M., Folwer, J.F., Hendry, J.H. & Scott, D. (eds) p. 731. Taylor and Francis: London.

CHAPLIN, D.C. & ACKER, B. (1987). Potentiation of RSU1069 tumour cytotoxicity by hydralazine: a new approach to selective therapy. *Int. J. Radiat. Oncol. Biol. Phys.*, **13**, 579.

CHAPLIN, D.T., DURAND, R.E. & OLIVE, P.L. (1986). Acute hypoxia in tumours: implications for modifiers of radiation effects. *Int. J. Radiat. Oncol. Biol. Phys.*, **12**, 1279.

DENEKAMP, J., HILL, S.A. & HOBSON, B. (1983). Vascular occlusion and tumour cell death. *Eur. J. Cancer. Clin. Oncol.*, **19**, 271.

DULHANTY, A.M., LI, M. & WHITMORE, G.F. (1989). Isolation of Chinese Hamster ovary cell mutants deficient in excision repair and mitomycin C bioactivation. *Cancer Res.*, **49**, 117.

DUNN, J.F., FROSTICK, S., ADAMS, G.E. & 4 others (1989). Induction of tumour hypoxia by a vasoactive agent. A combined NMR and radiobiological study. *FEBS Lett.*, **249**, 343.

HOBAN, P.R., WALTON, M.I., ROBSON, C.N. & 5 others (1990). Mitomycin C resistance under aerobic but not hypoxic conditions in a mammalian cell line: association with impaired drug activation and decreased NADPH: cytochrome P450 reductase activity. *Cancer Res.* (in the press).

HORSMAN, M.R., CHRISTENSEN, K.L. & OVERGAARD, J. (1989). Hydralazine induced enhancement of hyperthermic damage in a C3H mammary carcinoma *in vivo*. *Int. J. Hypertherm.*, **5**, 122.

KENNEDY, K.A., TEICHER, B.A.., ROCKWELL, S. & SARTORELLI, A. (1980). The hypoxic tumour cell: a target for selective cancer chemotherapy. *Biochem. Pharmacol.*, **29**, 1.

KEYES, S.R., FRACASSO, P.M., HEIMBROOK, D.C., ROCKWELL, S., SLIGAR, S.G. & SARTORELLI, A.C. (1984). Role of NAD(P)H: Cytochrome c reductase and DT-diaphorase in the biotransformation of mitomycin C. *Cancer Res.*, **44**, 5638.

KIRKPATRICK, D.L. (1989). The development of hypoxic tumour cell cytotoxic agents. *Pharmacol. Ther.*, **40**, 383.

KOPPER, L. & STEELE, G.G. (1975). The therapeutic response of three human tumor lines maintained in immune-suppressed mice. *Cancer Res.*, **35**, 2704.

MCNALLY, N.J., DENEKAMP, J., SHELDON, P.W., FLOCKHART, I.R. & STEWART, F.A. (1978). Radiosensitization by misonidazole (Ro-07-0582): the importance of timing and tumour concentrations. *Radiat. Res.*, **73**, 568.

MARSHALL, R.S., PATERSON, M.C. & RAUTH, A.M. (1989). Deficient activation by a human cell strain leads to mitomycin resistance under aerobic but not hypoxic conditions. *Br. J. Cancer*, **59**, 341.

O'NEILL, P., MCNEIL, S.S. & JENKINS, T.C. (1987). Mechanism of action of some bioreductive 2-nitroimidazoles: comparison of *in vitro* cytotoxicity and ability to induce DNA strand breakage. *Biochem. Pharmacol.*, **36**, 1787.

RAUTH, A.M., MOHINDRA, J.K. & TANNOCK, I.F. (1983). Activity of Mitomycin C for aerobic and hypoxic cells *in vitro* and *in vivo*. *Cancer Res.*, **43**, 4154.

RICE, G.C., HOY, C. & SCHIMKE, R.T. (1986). Transient hypoxia enhances the frequency of dihydrofolate reductase gene amplification in chinese hamster ovary cells. *Proc. Natl Acad. Sci. USA*, **83**, 5978.

STRATFORD, I.J. & ADAMS, G.E. (1982). Nitroimidazoles as hypoxia-mediated drugs in cancer chemotherapy. In *Nitroimidazoles Chemistry, Pharmacology and Clinical Applications*, NATO Advanced Study Institutes Series A: Life Sciences Vol. 42, Breccia, A., Rimondi, C. & Adams, G.E. (eds) p. 67. Plenum: London.

STRATFORD, I.J., ADAMS, G.E., GODDEN, J. & HOWELLS, N. (1989). Induction of tumour hypoxia post-irradiation: a method for increasing the sensitizing efficiency of misonidazole and RSU-1069 *in vivo*. *Int. J. Radiat. Biol.*, **55**, 411.

STRATFORD, I.J., ADAMS, G.E., GODDEN, J., HOWELLS, N., NOLAN, J. & TIMPSON, N. (1988). Potentiation of the anti-tumour effect of melphalan by the vaso-active drug, hydralazine. *Br. J. Cancer*, **58**, 122.

STRATFORD, I.J., GODDEN, J., HOWELLS, N., EMBLING, P. & ADAMS, G.E. (1987). Manipulation of tumour oxygenation by hydralazine increases the potency of bioreductive radiosensitizers and enhances the effect of melphalan in experimental tumours. In *Radiation Research* Vol. 2, Fielden, E.M., Fowler, J.F., Hendry, J.H. & Scott, D. (eds) p. 737. Taylor and Francis: London.

STRATFORD, I.J., O'NEILL, P., SHELDON, P.W., SILVER, A.R.J., WALLING, J.M. & ADAMS, G.E. (1986*a*). RSU-1069, a nitroimidazole containing an aziridine group: bioreduction greatly increases cytotoxicity under hypoxic conditions. *Biochem. Pharmacol.*, **36**, 105.

STRATFORD, I.J., WALLING, J.M. & SILVER, A.R.J. (1986*b*). The differential cytotoxicity of RSU-1069: cell survival studies indicating interaction with DNA as a possible mode of action. *Br. J. Cancer*, **53**, 339.

SUIT, H.D. & SHALEK, R.J. (1963). Response of spontaneous mammary carcinoma of the C3H mouse to X-irradiation given under conditions of local tissue anoxia. *J. Natl Cancer Inst.*, **31**, 497.

SUTHERLAND, R.M. (1974). Selective chemotherapy of non-cycling cells in an *in vitro* tumour model. *Cancer Res.*, **34**, 3501.

TANNOCK, I. & GUTTMAN, P. (1981). Response of Chinese hamster ovary cells to anti-cancer drugs under aerobic and hypoxic conditions. *Br. J. Cancer*, **43**, 245.

TEICHER, B.A., LAZO, J.S. & SARTORELLI, A.C. (1981). Classification of antineoplastic agents by their selective toxicities towards oxygenated and hypoxic cells. *Cancer Res.*, **41**, 73.

THOMLINSON, R.H. & GRAY, L.H. (1955). The histological structure of some human lung cancers and the possible implication for radiotherapy. *Br. J. Cancer*, **9**, 539.

TWENTYMAN, P.R., BROWN, J.M., GRAY, J.W., FRANKO, A.J., SCOLES, M.A. & KALEMAN, R.F. (1980). A new mouse tumour model system (RIF-1) for comparison of end-point studies. *J. Natl Cancer Inst.*, **64**, 595.

VARGHESE, A.J. & WHITMORE, G.F. (1983). Modification of Guanine derivatives by reduced 2-nitroimidazoles. *Cancer Res.*, **43**, 78.

WALLING, J.M., DEACON, J., HOLLIDAY, S. & STRATFORD, I.J. (1989). High uptake of RSU-1069 and its analogues into melanotic melanomas. *Cancer Chemother. Pharmacol.*, **24**, 28.

WALTON, M.I., WOLF, C.R. & WORKMAN, P. (1989). Molecular enzymology of the reductive bioactivation of hypoxic cell cytotoxins. *Int. J. Radiat. Oncol. Biol. Phys.*, **16**, 983.

WALTON, M.I. & WORKMAN, P. (1988). Pharmacokinetics and metabolism of the mixed function hypoxic cell sensitizer prototype RSU-1069 in mice. *Cancer Chemother. Pharmacol.*, **22**, 275.

WHITMORE, G.F. & GULYAS, S. (1986). Studies on the toxicity of RSU1069. *Int. J. Radiat. Oncol. Biol. Phys.*, **12**, 1219.

ZEMAN, E.M., BROWN, J.M., LEMMON, M.J., HIRST, V.K. & LEE, W.W. (1986). SR4233: a new bioreductive agent with high selective toxicity for hypoxic mammalian cells. *Int. J. Radiat. Oncol. Biol. Phys.*, **12**, 1239.

ZEMAN, E.M., HIRST, V.K., LEMMON, M.J. & BROWN, J.M. (1988). Enhancement of Radiation induced cell killing by the hypoxic cell toxin SR4233. *Radiother. Oncol.*, **12**, 209.